# Stat5-deficient hematopoiesis is permissive for Myc-induced B-cell leukemogenesis

**DOI:** 10.18632/oncotarget.5009

**Published:** 2015-08-22

**Authors:** Zhengqi Wang, Magdalena Medrzycki, Silvia T. Bunting, Kevin D. Bunting

**Affiliations:** ^1^ Department of Pediatrics, Division of Hematology-Oncology-BMT, Aflac Cancer and Blood Disorders Center of Children's Healthcare of Atlanta and Emory University, Atlanta GA; ^2^ Department of Pathology, Children's Healthcare of Atlanta, Atlanta GA

**Keywords:** hematopoiesis, leukemogenesis, lymphoid-primed multipotent progenitor, B-cell transformation, transcription factor

## Abstract

Despite being an attractive molecular target for both lymphoid and myeloid leukemias characterized by activated tyrosine kinases, the molecular and physiological consequences of reduced signal transducer and activator of transcription-5 (Stat5) during leukemogenesis are not well known. Stat5 is a critical regulator of mouse hematopoietic stem cell (HSC) self-renewal and is essential for normal lymphocyte development. We report that pan-hematopoietic deletion in viable adult Vav1-Cre conditional knockout mice as well as Stat5ab^null/null^ fetal liver transplant chimeras generated HSCs with reduced expression of quiescence regulating genes (Tie2, Mpl, Slamf1, Spi1, Cited2) and increased expression of B-cell development genes (Satb1, Dntt, Btla, Flk2). Using a classical murine B-cell acute lymphoblastic leukemia (B-ALL) model, we demonstrate that these HSCs were also poised to produce a burst of B-cell precursors upon expression of Bcl-2 combined with oncogenic Myc. This strong selective advantage for leukemic transformation in the background of Stat5 deficient hematopoiesis was permissive for faster initiation of Myc-induced transformation to B-ALL. However, once established, the B-ALL progression in secondary transplant recipients was Stat5-independent. Overall, these studies suggest that Stat5 can play multiple important roles that not only preserve the HSC compartment but can limit accumulation of potential pre-leukemic lymphoid populations.

## INTRODUCTION

The hematopoietic stem cell (HSC) compartment is divided into more quiescent self-renewing myeloid-biased and more actively cycling lymphoid-biased populations [1–4]. This antagonistic relationship between the self-renewal program and the earliest lymphoid lineage commitment appears to be an important checkpoint. Modulating lymphoid lineage priming has also been recently proposed as a novel method for promoting human HSC self-renewal and expansion [5]. c-Kit^+^lineage^neg^Sca-1^+^ (KLS) cells co-expressing Flt3 (Flk2) and Cd34 have a lymphoid-dominated short-term reconstitution potential and are referred to as lymphoid-primed multipotent progenitors (LMPP)s [6, 7] with limited megakaryocyte/erythroid lineage potential. Efforts to identify cell surface markers that permit separation of lymphoid vs. myeloid primed HSCs discovered that the signaling lymphocytic activation molecule family member 1 (Slamf1) [8] in conjunction with canonical HSC cell-surface markers [9], or with the cell capacity for Hoechst dye efflux [2, 10, 11], or the expression of endothelial protein C receptor (EPCR) [12], allows for prospective enrichment of HSC populations with distinct characteristics. Higher Cd150 expression in HSCs predicts robust HSC self-renewal and more potential for myeloid reconstitution while lower Cd150 expression in HSCs predicts more potential toward lymphoid reconstitution and marks the transition from self-renewal toward LMPPs [13]. Progressive increases of Slamf1 in aged HSCs are also associated with a higher ability for myeloid-dominated HSC self-renewal [3]. However the molecular regulation of Slamf1 in HSCs is not well characterized.

Early steps in leukemic evolution in a pre-leukemia state involve increased HSC fitness and clonal expansion. Interestingly, LMPP-like cells have been implicated as leukemia-initiating cells in acute myeloid leukemia (AML) [14] and chronic lymphocytic leukemia (CLL) [15], suggesting that pre-leukemic progenitors that are fit enough to expand and dominate niches could thrive in backgrounds that have reduced HSC fitness due to aging [16] or other factors. Signal transducer and activator of transcription-5 (Stat5) comprises two separate genes, Stat5a and Stat5b that are important regulators of normal HSC fitness [17–19]. Stat5 deficiency impairs long-term multilineage competitive repopulation capacity of HSCs, resulting in persistently more active cycling, increased apoptosis, and reduced long-term HSCs [20]. Stat5 is also generally recognized as a requisite driver of hematologic malignancy in cells expressing mutant receptor tyrosine kinases. However, in cases where leukemogenesis is initiated and propagated without Stat5 hyper-activating tyrosine kinase mutations, a potential role for Stat5 has not been considered. Stat5 has a tumor-suppressor function in hepatocellular carcinoma [21, 22] and IL-7/Stat5 has recently been shown to suppress expression of the B-cell mutator activation-induced cytidine deaminase (Aid) to protect against leukemic transformation [23]. Therefore we set out to determine the molecular regulation of HSCs by Stat5 and understand whether the context of defective hematopoiesis in the absence of Stat5 could predispose toward initiation or progression of a classic non-Stat5 dependent Eμ-Myc/H2K-Bcl-2 driven murine B-lymphoblastic leukemia (B-ALL).

## RESULTS

### Stat5-deficient HSCs are functionally impaired and have a lymphoid-biased phenotype

We have generated novel Stat5 knockout mice using Vav1-Cre which gives pan-hematopoietic deletion [24]. Although mice with embryonic deletion of Stat5 in all tissues have severe perinatal lethality, Vav1-Cre/+Stat5ab^fl/fl^ mice showed normal survival but with the diagnostic signs of Stat5 deficiency including anemia and lymphopenia (Supplementary Figure S1A–S1C; Supplementary Table S1). These mice have remained fully deleted into adulthood with deletion of the Stat5 genomic locus of 98% in sorted KLS cells. Competitive repopulation was 16-fold reduced with knockout (3.3 ± 0.7%; *n* = 5) compared to wild-type (55 ± 2%; *N* = 5) in a 1:1 mix with wild-type BoyJ (Supplementary Figure S1D). When Vav1-Cre/+Stat5ab^fl/fl^ mice were used as recipients in non-ablative transplantation, the percentage of total donor peripheral blood engraftment was very high in Vav1-Cre/+Stat5ab^fl/fl^ mice (Figure S1E). High levels of donor multilineage engraftment were also observed in Vav1-Cre/+Stat5ab^fl/fl^ mice (Supplementary Figure S1E), like in our prior study with Mx1-Cre/+Stat5ab^fl/fl^ mice [20], indicating that strong selective pressure for normal hematopoietic stem/progenitor expansion in the Stat5-deficient background.

**Figure 1:**
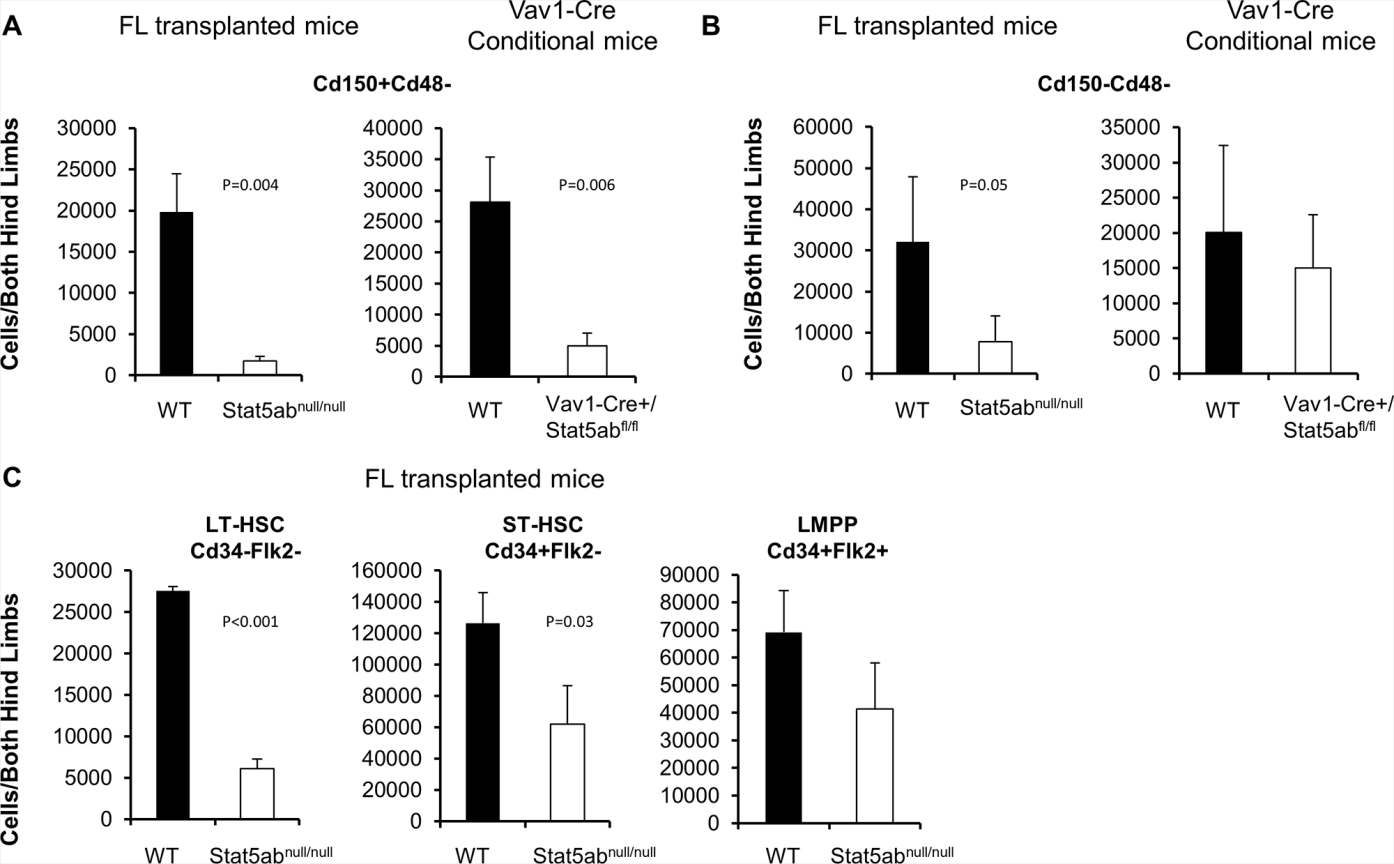
Stat5-deficient HSCs have a lymphoid-biased phenotype with greatly reduced long-term HSCs Bone marrow cells from either Vav1-Cre+/Stat5ab^fl/fl^ and littermates or wild-type and Stat5ab^null/null^ fetal liver transplanted chimeric mice were assayed by multi-parameter flow cytometry to quantitate primitive HSC populations. Three separate experiments were performed with 3 to 5 mice per genotype compared. The absolute number of primitive HSC populations in bone marrow cells from both hind limbs is presented. **A–B.** Bone marrow was stained with antibodies against lineage markers, c-Kit, Sca-1, Cd150 and Cd48 antibodies. For fetal liver transplanted chimeric mice the donor Cd45.2 antibody was also included. **C.** Bone marrow isolated from fetal liver transplanted chimeric mice was analyzed by flow cytometry with antibodies against lineage markers, c-Kit, Sca-1, Cd34, and Flk2.

To determine the role of Stat5 in adult hematopoietic stem/progenitor cell heterogeneity, bone marrow cells were obtained from Stat5ab^null/null^ fetal liver transplanted mice or Vav1-Cre conditional deletion staining with either the combination of the Slam markers Cd150/Cd48 or Cd34/Flk2 in addition to the KLS markers. Even though the total bone marrow cellularity was reduced about 40%, the deletion of Stat5 led to significantly reduced absolute numbers of LT-HSC (Cd150^+^Cd48^−^KLS) (Figure 1A) but smaller decreases in ST-HSC (Cd150^−^Cd48^−^KLS) (Figure 1B). Using Cd34/Flk2 markers for LT-HSC (Cd34^−^Flk2^−^KLS) and ST-HSC (Cd34^+^Flk2^−^KLS), similar results were obtained (Figure 1C). The LMPP fraction (Cd34^+^Flk2^+^KLS) was not changed. These results suggested that Stat5-deficient HSC/HPC may become skewed toward a lymphoid-biased phenotype, possibly to maintain the LMPP pool for a deficient absolute number of LT-HSCs.

**Figure 2:**
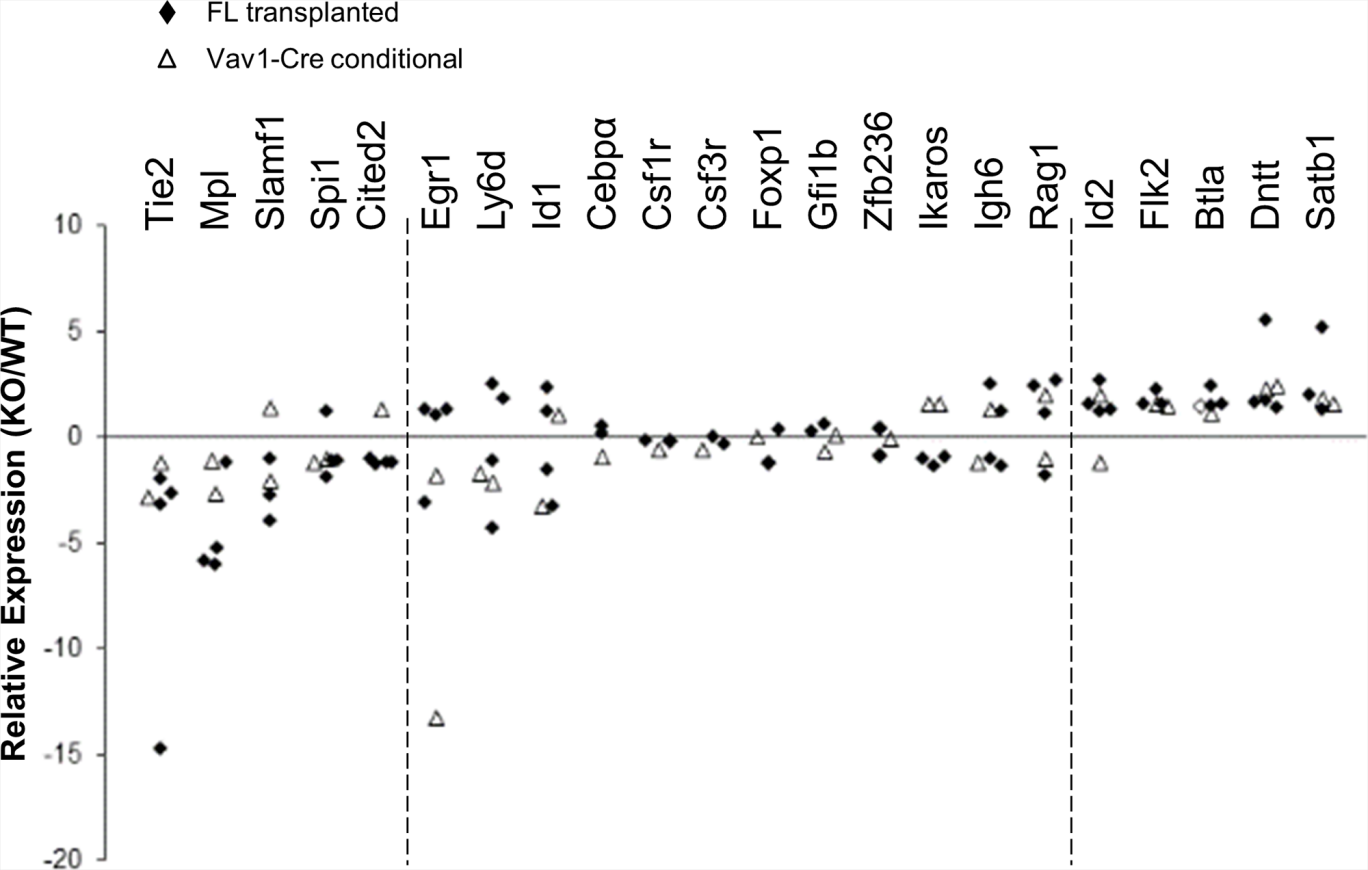
Stat5-deficient HSCs have reduced expression of HSC/quiescence associated genes and increased expression of lymphoid-lineage genes Bone marrow cells from either Vav1-Cre+/Stat5ab^fl/fl^ and littermates or wild-type and Stat5ab^null/null^ fetal liver transplanted chimeric mice were collected and the KLS fractions were isolated by flow cytometry sorting. Real-time PCR analysis of purified KLS from wild-type or Stat5ab^null/null^ fetal liver transplanted mice (marked as solid diamonds) or KLS from Stat5ab^fl/fl^ and Vav1-Cre+/Stat5ab^fl/fl^ mice (open triangles) is presented as fold changes of individual gene expression between Stat5ab^null/null^ and wild-type KLS cells after the normalization to GAPDH using the ΔΔCt method. The primer sets analyzed are shown on the top with the decreased transcripts shown to the left and the increased transcripts shown to the right. The middle primer sets were not significantly changed.

**Figure 3:**
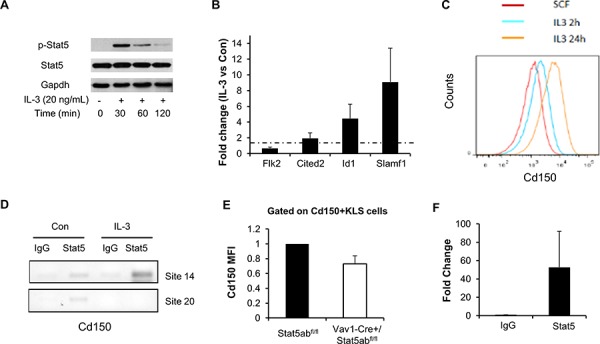
Stat5 activation directly regulates the myeloid differentiation marker Slamf1 in EML C1 and primary KLS cells **A.** EML C1 cells were switched from maintenance culture media (SCF alone) to differentiation culture media (SCF + IL-3) and phosphorylation of Stat5 was determined by Western blotting in cells grown under both conditions. **B.** Real-time PCR analysis of EML C1 cells 2 h after *in vitro* IL-3 exposure. Data presented (averages of four separate experiments) are fold changes of genes in IL-3 treated vs. SCF alone after the normalization to GAPDH control using ΔΔCt method. Error bars represent standard deviation. **C.** Staining for Cd150 in EML C1 cells after 2 h and 24 h *in vitro* IL-3 exposure. Cells were gated on Cd150^+^KLS cells. **D.** Stat5 was immunoprecipitated from formaldehyde-cross-linked lysates prepared from EML C1 cells treated with or without IL-3 for 2 h. Binding of Stat5 to putative Stat5 consensus binding sites 14 and 20 (control) in the Slamf1 gene was determined by PCR amplification of immunoprecipitated DNA. Nonspecific binding was assayed by rIgG isotype-control immunoprecipitation of cross-linked lysates. Results are representative of 2 independent experiments. **E.** Mean fluorescence intensity (MFI) of Cd150 in Cd150^+^KLS of Vav1-Cre/+Stat5ab^fl/fl^ and Stat5ab^fl/fl^ mice. Results are the average from four independent experiments and were normalized to Cd150 MFI for Stat5ab^fl/fl^ mice. **F.** Stat5 was immunoprecipitated from formaldehyde-cross-linked lysates prepared from about 1 million sorted wild type mice KLS cells. Binding of Stat5 to putative Stat5 consensus binding site 14 in the Slamf1 gene was determined by real time PCR amplification of immunoprecipitated DNA (*n* = 3, *p* = 0.08).

We next checked expression of a series of selected genes associated with either HSC quiescence/self-renewal or lymphoid lineage development. Real-time PCR on fetal liver transplanted or Vav1-Cre/+Stat5ab^fl/fl^ KLS cells was performed and the result is shown in Figure 2. HSC related genes Tie2, Mpl, Slamf1, Spi1 (Pu.1), and Cited2 were significantly reduced while lymphoid priming associated genes Flk2, Btla, Dntt, and Satb1 were significantly increased. Other genes that were analyzed did not show significant changes. We next wanted to find out whether any of the regulated genes were direct Stat5 transcriptional targets. To do this, we first utilized multipotent erythroid-myeloid-lymphoid (EML) C1 cells [25] as a screening tool to help focus on candidate targets for chromatin immunoprecipitation (ChIP) in primary KLS cells. EML cells maintained in stem cell factor (SCF) alone showed no Stat5 activation. Interleukin (IL)-3 treatment quickly induced Stat5 activation (Figure 3A) and two hours after treatment the expression of Slamf1, Id1, and Cited2 was increased 9-fold, 5-fold, and 2-fold respectively while Flk2 was decreased 2-fold (Figure 3B). As expected, this pattern of change was inverse from that seen following Stat5 deletion in Stat5ab^null/null^ fetal liver transplanted or Vav1-Cre/+Stat5ab^fl/fl^ KLS cells. Flow cytometry analysis showed the mean fluorescence intensity (MFI) of Slamf1 was subsequently increased in the EML C1 cells following IL-3 treatment compared to SCF treatment alone in a time-dependent manner (Figure 3C).

Searching for the conserved STAT5 binding site (TTCnnnGAA) in Slamf1, Cited2, Flk2, Id1, and Satb1 genes, we found 3, 1, 1, 1, and 8 sites respectively that are conserved between mice and humans (Supplementary Table S2). Chromatin immunoprecipitation analysis showed that Stat5 binds to the conserved Stat5 binding site #14 in the Slamf1 gene in IL-3 treated EML C1 cells (Figure 3D). In primary mouse bone marrow cells, wild type Cd150^+^KLS had a higher Cd150 MFI compared to Stat5-deficient Cd150^+^KLS cells (Figure 3E) and Stat5 bound specifically to Slamf1 site 14 in sorted wild type bone marrow KLS cells (Figure 3F). Additional binding sites in Cited2, Flk2, and Satb1 were tested but no evidence for direct binding was observed at these conserved sites in EML cells. Id1 has previously been documented as a Stat5 direct target gene [26]. The expression of Id1 was also induced in the lineage depleted primary bone marrow cells in response to IL-3 treatment (data not shown).

### Bcl-2 rescues Stat5-deficient lymphoid progenitors and synergizes with Myc to promote massive expansion

**Figure 4:**
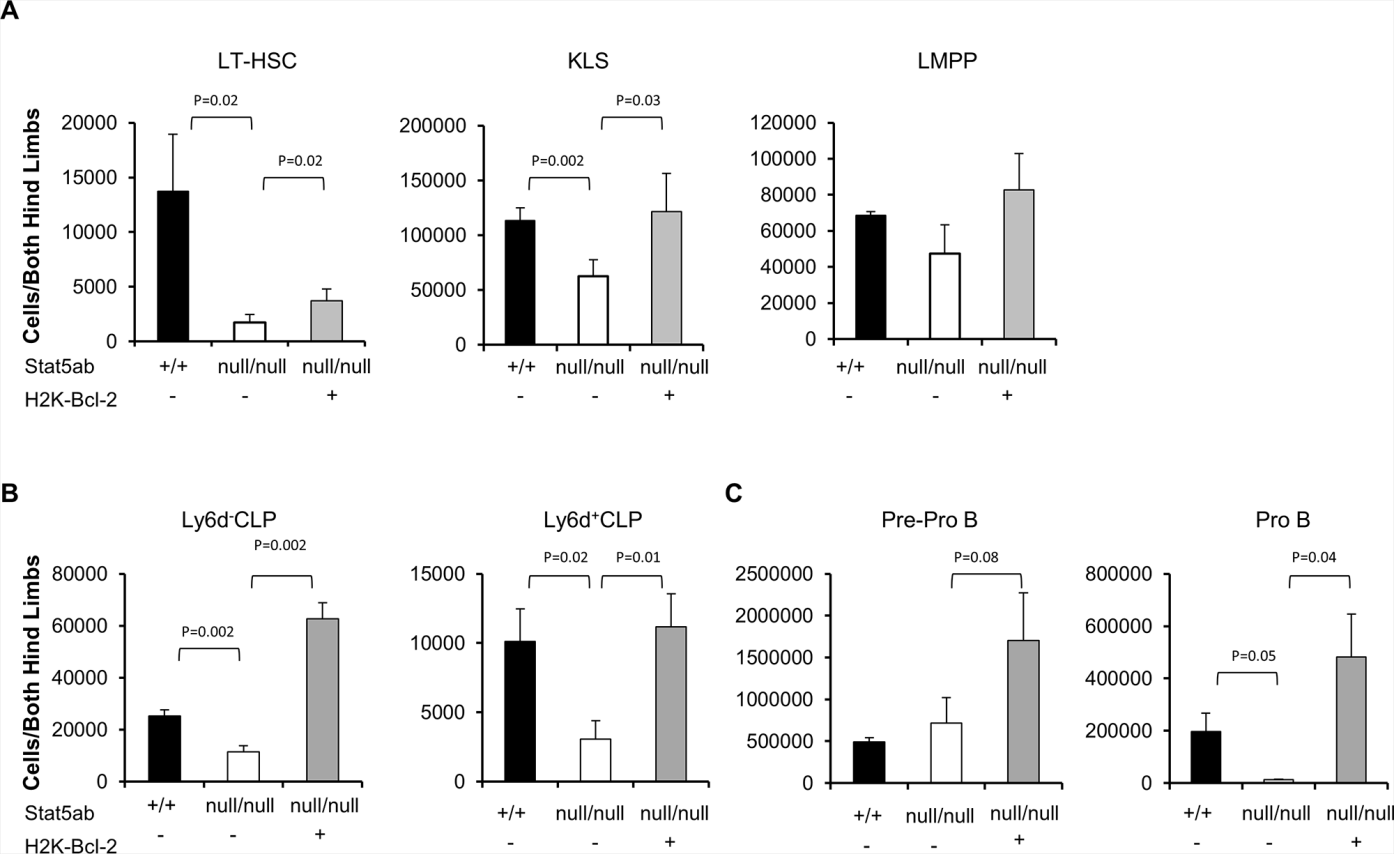
Rescue of Stat5-deficient bone marrow lymphoid progenitors by transgenic Bcl-2 expression Wild-type, Stat5ab^null/null^, and H2K-Bcl-2/Stat5ab^null/null^ fetal liver cells were transplanted into lethally irradiated BoyJ mice. 12–16 weeks after the transplantation, bone marrow cells were stained with antibodies against lineage markers, c-Kit, Sca-1, Flk2, Cd34 and Cd45.2 antibodies. Two independent transplantation experiments were performed with 3–5 mice per group. **A.** The absolute number of Cd34^−^Flk2^−^ KLS (LT-HSC), KLS, and Cd34^+^Flk2^+^ KLS (LMPP) populations in bone marrow cells from both hind limbs are presented. **B.** The absolute number of Ly6d^−^CLP and Ly6d^+^CLP **C.** The absolute number of Pre-pro B and Pro B cell populations in the bone marrow cells obtained from both hind limbs are presented. CLP is defined as IL-7R^+^c-Kit^med^Sca-1^med^Lineage^−^. Pre-pro B cells are c-Kit^+^B220^+^Cd19^−^IgM^−^ and Pro B cells are c-Kit^+^B220^+^Cd19^+^IgM^−^.

**Figure 5:**
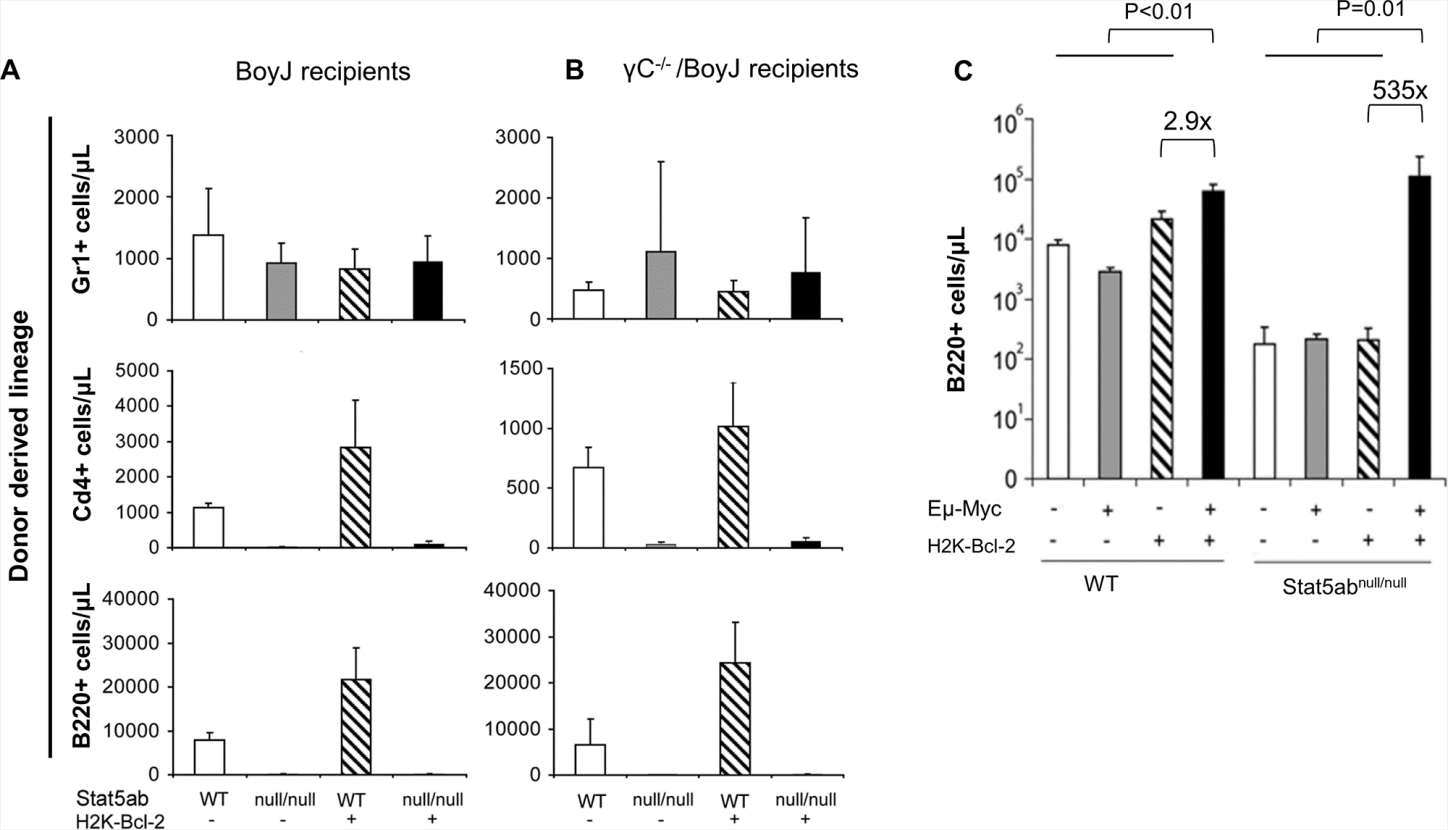
Expansion of peripheral blood Stat5-deficient B-cell precursors by the combined expression of Myc and Bcl-2 Flow cytometry analysis of B-, T-, and myeloid cell development in Stat5ab^null/null^ fetal liver transplanted recipients alone or combined with transgenic H2K-Bcl-2 expression. E14.5 fetal liver cells (wild-type (WT), Stat5ab^null/null^ (null/null), H2K-Bcl-2, or H2K-Bcl-2/Stat5ab^null/null^) were obtained from timed mating of H2K-Bcl-2/Stat5ab^+/null^ x Stat5ab^+/null^ genetic crosses. All donor mice used for these experiments were backcrossed to the C57BL/6 background and were transplanted into lethally irradiated Boy J recipients **(A)** or lethally irradiated common γC^−/−^ mice (BoyJ background) **(B)** at a ratio of 1 donor fetal liver to 5 recipient mice. 16 weeks after the fetal liver transplantation, recipient mouse peripheral blood was analyzed for the percentage of donor derived cells in the myeloid lineage (Gr1^+^), T-cell lineage (CD4^+^) and B-cell lineage (B220^+^) along with the donor CD45.2 marker. The absolute numbers of donor-derived leukocytes (Gr-1^+^, B220^+^, CD4^+^) in each lineage are represented. Results are combined from two independent fetal liver transplantation experiments (total 10 recipient mice per genotype per recipient background). C. Wild-type, Stat5ab^null/null^, and Stat5ab^null/null^ +/− Myc/Bcl-2 transgenic fetal liver cells were transplanted into lethally irradiated BoyJ mice. Four weeks after the transplantation, mice were analyzed for peripheral blood hematology. B220+ cells from transplanted mice 4 weeks after transplantation (*n* = 5 for each genotype) showed a dramatic increase in the absence of Stat5.

Since we observed strong selective pressure for normal HSC repopulation in Stat5-deficient hosts, we next asked whether the LMPPs lacking Stat5 were poised for rapid expansion of the precursor B-lymphocyte pool. To do this, Stat5-deleted mice were crossed with transgenic mice expressing Bcl-2 and/or Myc. In the peripheral blood, Bcl-2 alone was not sufficient to produce B220^+^ or mature IgM^+^B220^+^ cells (data not shown) consistent with reports that Rag1-Cre Stat5 conditional knockout mice with transgenic Bcl-2 cannot develop past the pro-B-cell stage [27]. With bone marrow fractions we analyzed KLS cells, Cd34^−^Flk2^−^KLS (LT-HSC), and Cd34^+^Flk2^+^ KLS (LMPP) following the presence or absence of transgenic Bcl-2 expression. Interestingly, Bcl-2 expression was sufficient to correct the number of KLS cells (Figure 4A) with the notable exception of LMPPs which were already increased in Stat5 knockout mice even without Bcl-2 expression. We analyzed the common lymphoid progenitor (CLP) along with Ly6d to separate CLP into the all-lymphoid (Ly6d^−^CLP) or the B-cell biased lymphoid progenitor (Ly6d^+^CLP) [28] (Figure 4B). The number of CLPs was reduced in the absence of Stat5. However, Bcl-2 expression was sufficient to restore both subsets of CLPs. Pre-pro B (c-Kit^+^B220^+^Cd19^−^IgM^−^) and Pro B (c-Kit^+^B220^+^Cd19^+^IgM^−)^ cells in the bone marrow compartment were also analyzed. In the absence of Stat5, there was a slight increase in number of Pre-pro B cells but very little Pro B cells. With the expression of Bcl-2 transgene, both populations of cells were significantly increased compared to the wild-type control (Figure 4C). This is consistent with previous report that deficiency of Stat5 blocks B-cell development at a Pre-pro B cell stage [27]. Therefore, we co-expressed Eμ-Myc, which does not require Stat5 for oncogenesis [29], but can provide complementary signals with Bcl-2 to drive B-cell acute lymphoblastic leukemia in wild-type mice (Supplementary Figure S2). We found that Bcl-2 alone was not sufficient to restore the number of circulating B220^+^ B-cells (Figure 5A/5B). However, Bcl-2 combined with Myc expression caused a mild anemia (data not shown) in a genotype dependent manner and induced robust B220^+^ B-cell development with over a 3-log increase in number compared to Stat5 knockout alone (Figure 5C) measured one month post-transplantation. This expansion and fold increase in the total white blood cell count was 184-fold greater in the Stat5 knockout relative to the wild-type when each was compared to its own H2K-Bcl-2 control (*P* < 0.01 for each).

### Stat5 suppresses Myc/Bcl-2 driven B-cell leukemia initiation but not progression

**Figure 6:**
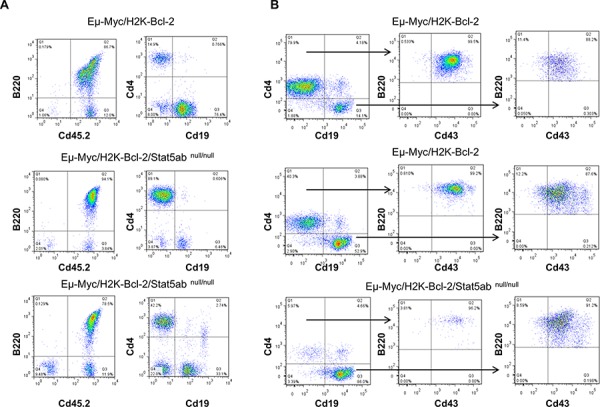
Myc-induced B-cell transformation occurs with developmental blocks at comparable stages in both wild-type and Stat5-deficient leukemia E14.5 fetal liver cells (Eμ-Myc/H2K-Bcl-2 or Eμ-Myc/H2K-Bcl-2/Stat5ab^null/null^) on the C57BL/6 background obtained from timed matings as described in Supplementary Figure S2 were transplanted into lethally irradiated BoyJ recipient mice at the ratio of 1 donor fetal liver to 5 recipient mice. Four weeks after the fetal liver transplantation, mouse peripheral blood was analyzed for B-cell development with CD4, CD19, CD43, B220, and donor-specific CD45.2 antibodies. **A.** Representative histograms are shown from 5 mice of either Eμ-Myc/H2K-Bcl-2 or Eμ-Myc/H2K-Bcl-2/Stat5ab^null/null^ fetal liver transplanted mice. Gating strategy on live cell populations; Eμ-Myc/H2K-Bcl-2 fetal liver cells mainly showed pro-B phenotypes (CD4^−^Cd19^+^Cd43^+^B220^+^) and Eμ-Myc/H2K-Bcl-2/Stat5ab^null/null^ showed either pre-pro-B (CD4^+^CD19^−^CD43^+^B220^+^) or mixed pre-pro-B and pro-B phenotype. **B.** Representative histogram from a separate experiment from 4 Eμ-Myc/H2K-Bcl-2 or 3 Eμ-Myc/H2K-Bcl-2/Stat5ab^null/null^ fetal liver transplanted mice. Eμ-Myc/H2K-Bcl-2 fetal liver cells showed mixed pre-pro-B and pro-B phenotype while Eμ-Myc/H2K-Bcl-2/Stat5ab^null/null^ mice had mainly pro-B phenotypes.

**Figure 7:**
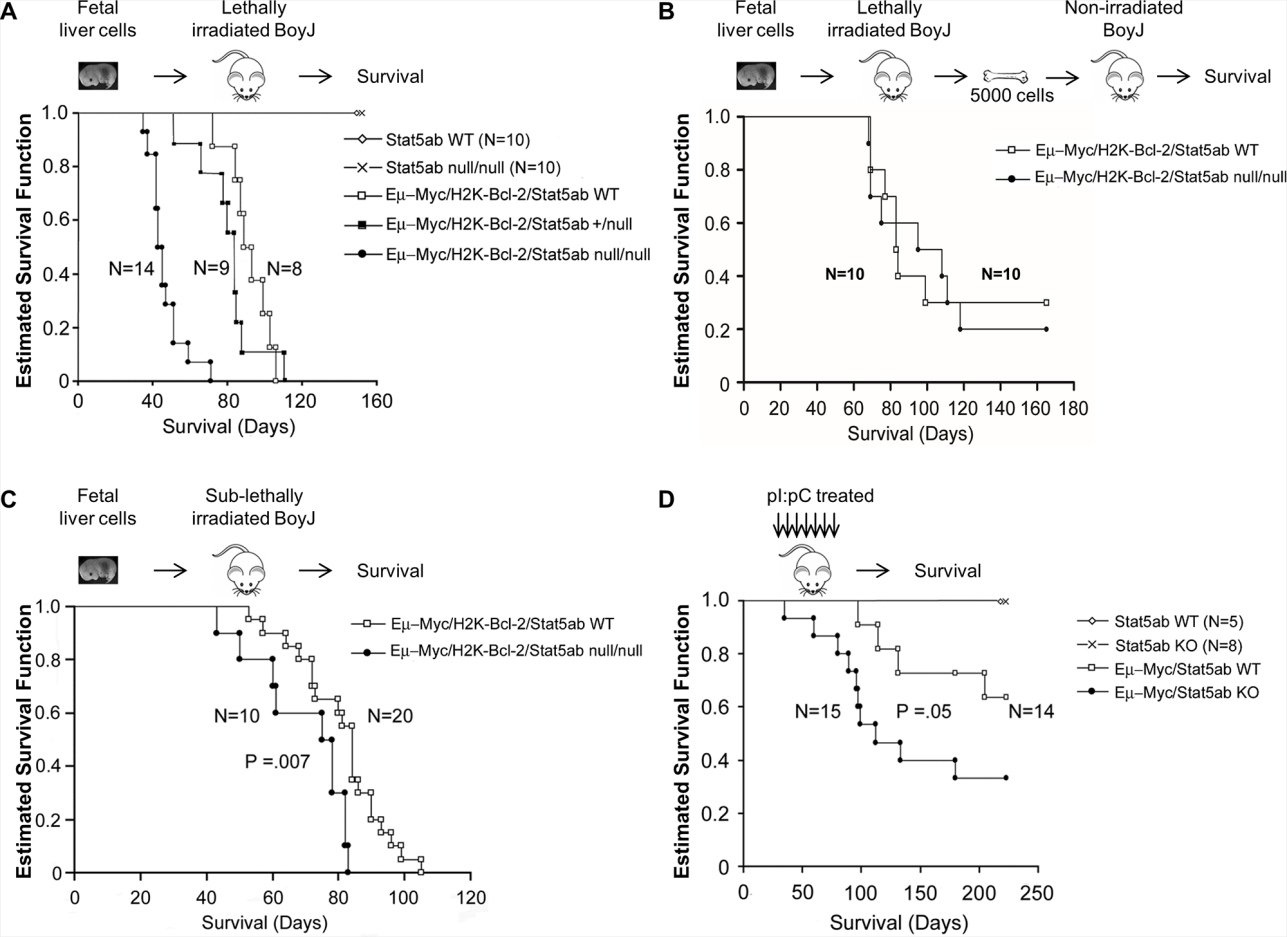
Faster initiation but not progression of B-ALL in the absence of Stat5 **A.** Kaplan-Meier survival analysis of lethally-irradiated (1100 cGy) transplanted mice. E14.5 wild-type, Stat5ab^null/null^, or Eμ-Myc/H2K-Bcl-2 combined with wild-type, heterozygous, or homozygous Stat5ab^null/null^ fetal liver cells were transplanted into lethally irradiated BoyJ mice. Data is combined from three sets of independent transplantations (*p* < 0.001). **B.** Non-irradiated secondary transplants were set up from the bone marrow of mice following the development of B-ALL and 5000 bone marrow cells per recipient was used for the transplantation. Secondary disease was monitored by Kaplan-Meier survival curve. Results are combined from two sets of independent transplantations. **C.** Sub-lethally (400 cGy) irradiated mice were transplanted with E14.5 wild-type or Stat5ab^null/null^ fetal liver cells to assess the impact of Myc/Bcl-2 on B-ALL development. Survival is shown as a Kaplan-Meier survival curve. **D.** Non-irradiated mice expressing transgenic Myc were monitored long-term for development of B-cell lymphoma and survival. Mice around 4 weeks old were injected every other day with 7 doses of 16 mg/kg pI:pC and monitored for long-term survival. Mice categorized as the wild-type group included wild-type cells as well as Stat5ab^fl/fl^ and Stat5ab^fl/+^ that lacked Cre recombinase expression. The knockout group of mice included both Mx1-Cre/+Stat5ab^fl/null^ and Mx1-Cre/+Stat5ab^fl/fl^ mice.

In the absence of Stat5, addition of transgenic Myc and Bcl-2 expression permitted expansion of B220^+^ B-cell precursors. In all experiments, the disease diagnosis and immunophenotype (B220, CD43, CD19, CD4) in the absence of STAT5 was comparable to wild-type, characterized by blocks at the pre-pro and pro-B cell stages (Figure 6A/6B, Supplementary Figure S3) with some variation in the proportions from mouse to mouse. We next wanted to determine whether the faster early B-cell development corresponded with a shorter latency of development of B-ALL. Strikingly, the increased B-lineage commitment translated into faster death in lethally-irradiated recipients of transplanted STAT5ab^null/null^ fetal liver cells (Figure 7A) but only a trend toward faster death with transplanted STAT5ab^+/null^ fetal liver cells. In contrast, secondary transplantation of equal numbers of bulk bone marrow cells from mice that had already developed leukemia into non-irradiated hosts showed equivalent disease progression with or without Stat5 expression (Figure 7B). Therefore, the skewing of hematopoiesis was predisposing to leukemia but not changing the overall leukemia potential of transformed cells. To eliminate concerns about the lethally-irradiated primary recipients, we also tested B-ALL development in sub-lethally irradiated mice receiving transplanted fetal liver cells (Figure 7C). While the difference in latency was not as large as in the irradiated recipients, there was still a significantly faster death rate in the transplanted mice. Finally, to completely eliminate the need for irradiation, we performed experiments using the Mx1-Cre/+Stat5ab^fl/fl^ mice crossed with the Myc transgene (Figure 7D). Four to five week old Eμ-Myc/Stat5ab^fl/fl^ mice along with control mice were treated with 7 doses of pI:pC injection every other day to delete Stat5. Although the latency of Myc-induced lymphoma was longer than in the more aggressive Myc/Bcl-2 B-ALL model, the results were also very striking, showing a considerably shorter time to death in mice lacking Stat5 expression. To confirm deletion of Stat5 in the pI:pC treated Stat5 conditional mice with lymphoma, CD43+/B220+ cell populations were sorted from either bone marrow or enlarged lymph nodes. Real-time PCR with genomic DNA confirmed consistent deletion of the floxed Stat5 locus (*n* = 3).

## DISCUSSION

Our studies focused on highly defined murine hematopoietic stem/progenitor subsets to assess the impact of Stat5 on gene expression regulating HSC heterogeneity and early differentiation decisions. We found that Stat5 regulates multiple critical regulators of early hematopoiesis such as Mpl, Spi1, Cited2, Satb1. Microarray studies by others have previously identified Stat5 target genes in human model systems that include, Gata1 [30], Cebpα [31], and Hif2α [32]. However, most of the effects of Stat5 on dysregulated genes in our study appear to be indirect, except for Slamf1 which was the only gene with potential conserved consensus binding sites that we could document specific binding in KLS cells. Stat5 activation of Slamf1 raises the question of whether this regulation is a general feature common to other cell types, since Slamf1 is a self-ligand cell surface glycoprotein expressed on T-cells, B-cells, macrophages, and dendritic cells. To date there have been very few studies of ChIP on primary KLS cells due to sensitivity and cost considerations. Several studies using KLS cells have focused on epigenetic histone protein modifications [33, 34] due to the abundance of their expression and the important role in regulation of gene expression. However, a recent study performed ChIP-seq on Cepbα null KLS cells using a commercially available linear amplification method [35] thus demonstrating feasibility of this approach for low abundance transcription factors in rare cells.

In the setting of oncogene expression (Myc/Bcl-2), Stat5 knockout caused massive expansion of B220^+^ B-cell precursors in the peripheral blood. Comparable selection for host cells expressing low levels of phosphorylated STAT5 has been reported in a BCR-ABL mouse model to permit a selective advantage for BCR-ABL expressing pro-B cells but not myeloid leukemia cells [16] over aged progenitors with declining competitive repopulating ability. Stat5 is well described as a molecular regulator of lymphoid and myeloid leukemias and myeloproliferative neoplasms driven by activated tyrosine kinase signaling [36–41] and deleting Stat5 has been shown to be therapeutic for these neoplasms [42–47]. However, we show here that Stat5 deletion has distinct consequences on early lineage commitment that could potentially create a favorable environment for survival and expansion of oncogene expressing pre-leukemic cells. In our experimental model for Myc/Bcl-2 driven B-ALL, the consequence was increased initiation of disease. In humans Myc/Bcl-2 represent the poor prognosis “double hit” lymphomas, thus differing from the disease manifestation in mice. However, our study showed similar reduced disease latency with either Myc alone (lymphoma) or Myc/Bcl-2 (leukemia). Importantly, as would be expected for this non-kinase driven leukemia, Stat5 expression or activation was not involved in the subsequent progression of established disease. This result is consistent with mouse model studies that showed that Stat5 is required for Bcr-Abl induced myeloid disease progression but that Stat5 deletion could not prevent progression to lymphoid blast crisis [45, 47, 48]. Additionally relapse is very high in Ph+ ALL patients following tyrosine kinase inhibitor therapy [49].

The murine Myc/Bcl-2 model, like human B-ALL, does not appear to be characterized by a stem cell hierarchy [50], so expansion of B-cell precursors downstream of the LMPP and expressing Bcl-2 would be expected to promote initiation of disease in combination with Eμ-Myc expression (2^nd^ hit). Likewise, Stat5 may directly impact pre-pro-B cells through tight regulation of the zinc finger transcriptional repressor Bcl6 [51] or Aid [23]. Here we show for the first time that Myc-driven B-ALL can be accelerated by Stat5 deletion. This finding could be of potential clinical significance associated with relapse by new clones as has been described following chemotherapy [52, 53] and future studies will focus on this question. In conclusion, this work provides a new facet to the regulation of lineage commitment by Stat5 and has potential implications for understanding the biology of human leukemia evolution.

## MATERIALS AND METHODS

### Mice

Mx1-Cre, Eμ-Myc, and γC^−/−^ mice on the C57BL/6 background (Cd45.2) as well as the B6.SJL-Ptprc^a^Pep3^b^/BoyJ (Cd45.1) congenic strain were obtained from the Jackson Laboratory (Bar Harbor, ME). Stat5ab^flox/+^ mice [54] were obtained from Lothar Hennighausen (NIH, Bethesda, MD) and were backcrossed to the C57BL/6 background more than 9 generations. Vav1-Cre mice [24] were from Thomas Graf (Center for Genomic Regulation, Barcelona). C57BL/6 background H2K-Bcl-2 transgenic mice were obtained from Jos Domen [55]. All mouse studies were approved by the Institutional Animal Care and Use Committee at Emory University (Atlanta, GA).

### Fetal liver and bone marrow cell transplantation

Wild-type and Stat5ab^null/null^ fetal liver transplanted chimeric mice were generated by transplanting E14.5 fetal livers (obtained from timed mating of Stat5ab^+/null^ X Stat5ab^+/null^ mice) with 1 donor fetal liver into 5 lethally irradiated recipients. H2K-Bcl-2/Stat5ab^null/null^ fetal liver transplanted chimeric mice were generated with E14.5 fetal livers from timed mating of H2K-Bcl-2/Stat5ab^+/null^ X Stat5ab^+/null^ mice. Eμ-Myc/H2K-Bcl-2/Stat5ab^null/null^ or wild-type fetal liver transplanted mice were generated with E14.5 fetal livers from timed mating of H2K-Bcl-2/Stat5ab^+/null^ X Eμ-Myc/Stat5ab^+/null^ or Eμ-Myc/Stat5ab^+/null^ X H2K-Bcl-2/STAT5ab^+/null^ mice. All recipient mice were conditioned with either 1100 cGy (lethal) or 400 cGy (sub-lethal) from a ^137^Cs source. Adult 4–5 week old Mx1-Cre conditional mice were treated with 16 mg/kg poly(I)-poly(C) (pI:pC) every other day for a total of 7 doses.

## SUPPLEMENTAL METHODS TABLES AND FIGURES


