# Supplementary material for: Stat5-deficient hematopoiesis is permissive for Myc-induced B-cell leukemogenesis
Source: Oncotarget. 2015 Aug 22;6(30):28961–72. doi: 10.18632/oncotarget.5009 (PMC4745704; doi:10.18632/oncotarget.5009)
Supplement: Supplementary file 1 [file oncotarget-06-28961-s001.pdf]

SUPPLEMENTAL METHODS

Cell culture

EML C1 cells were cultured in IMDM 20% horse serum, 1% antibiotic-antimycotic (Gibco by Life Technologies) 15% SCF conditioned medium from BHK/MKL cells or 50–100 ng/mL of recombinant murine SCF. $\gamma C^{-/-}$ mice were crossed to the Cd45.1 (BoyJ) background.

Non-ablated mouse transplantation

Non-ablated transplantation was carried out by the injection of a total of 5 million bone marrow cells via the lateral tail vein into non-conditioned recipients as previously described. Engraftment was determined by staining peripheral blood leukocytes with both Cd45.1 (donor) and Cd45.2 (host) antibodies.

Hematology and flow cytometry

Peripheral blood was obtained following puncture of either the facial vein or the retroorbital venous sinus. Blood was collected in a heparinized tube. Mouse hematology was determined using a HemaTrue Hematology Analyzer (Heska). Flow cytometry was performed on LSRII and cell sorting was performed on BD FACS Aria II cell sorter (BD Biosciences) at the Emory University Pediatrics Flow Cytometry Core. Antibodies for flow cytometry were obtained from either BD Biosciences (San Diego) or eBiosciences (San Diego). Data were analyzed using Flowjo software (TreeStar).

Chromatin immunoprecipitation (ChIP) assay

ChIP assay was carried out as previously described. Cells were treated with freshly made 1.6% formaldehyde in PBS and followed by the addition of glycine to a final concentration of 0.14 M. Cells were then washed twice with ice-cold PBS and resuspended in radio-immunoprecipitation assay lysis buffer (RIPA) containing protease and phosphatase inhibitors (Roche). Genomic DNA was sheared to lengths of approximately 0.4 to 1 kb fragments by sonicating the cell lysate (Fisher F60 Sonic Dismembrator). The chromatin supernatant was pre-cleared by adding rabbit IgG bound protein agarose for 1 h at 4°C and then the supernatant was incubated with Stat5 or control IgG antibody (C-17, Santa Cruz Biotechnology) along with protein A agarose at 4°C overnight. The pellet was then washed twice sequentially with RIPA buffer, 4 times with IP wash buffer (100 mM Tris pH 8.5, 500 mM LiCl, 1% NP-40, 1% deoxycholic acid), then two more times with RIPA buffer. Reversal of cross-linking was performed by adding crosslink reversal buffer (125 mM Tris pH 6.8, 10% β-mercaptoethanol, 4% SDS) at 99°C for 30 min. Finally, DNA was purified by phenol/chloroform extraction and followed by ethanol precipitation with carrier glycogen. For ChIP with EML C1 cells, 1 mg cross-linked lysates was used. For ChIP with wild-type KLS cells, around 1 million KLS were sorted from ~25 wild type mice for each experiment.

SUPPLEMENTARY TABLES AND FIGURES

Supplementary Table S1: Peripheral blood hematology of Vav1-Cre STAT5 conditional mice

	WBC*** $10^3/\mu L$	LYM*** $10^3/\mu L$	MONO*** $10^3/\mu L$	GRAN*** $10^3/\mu L$	HCT*** %	MCV** fl
STAT5ab fl/fl (*n* = 27)	8.46 ± 2.26	6.83 ± 1.73	0.50 ± 0.20	1.13 ± 0.74	47.1 ± 3.2	46.2 ± 1.6
Vav1-Cre/+ STAT5ab fl/fl (*n* = 17)	1.84 ± 1.01	1.11 ± 0.89	0.26 ± 0.12	0.48 ± 0.28	33.2 ± 4.3	44.1 ± 2.6
	RDWa** fl	HGB*** g/dL	MCHC g/dL	MCH* pg	RBC*** 106/µL	PLT*** 103/µL
STAT5ab fl/fl (*n* = 27)	33.3 ± 3.1	16.7 ± 1.2	35.5 ± 0.8	16.4 ± 0.36	10.2 ± 0.8	504 ± 103
Vav1-Cre/+ STAT5ab fl/fl (*n* = 17)	30.4 ± 2.9	11.9 ± 1.55	35.8 ± 0.55	15.8 ± 0.98	7.5 ± 0.78	379 ± 109

Notes:
1) * P value < 0.05 ** p value < 0.01 *** p value < 0.001
2). WBC: white blood cells; LYM: lymphocytes; MONO: monocytes; GRAN: granulocytes; HCT: hematocrit; MCV: mean corpuscular volume; RDW: Red cell distribution width; HGB: hemoglobin; MCHC: mean corpuscular hemoglobin concentration; MCH: mean corpuscular hemoglobin; RBC: red blood cells; PLT: platelets

Supplementary Table S2: The position of conserved Stat5 consensus binding sites (TTCnnnGAA) in selected potential Stat5 regulated genes

site	position	notes
**Slamf1 (total 21 sites in mouse and 23 sites in human)**		
5th site	1124 to 1133	conserved
8th site	6928 to 6937	conserved
14th site	21603 to 21712	conserved
20th site	35151 to 35160	non-conserved; control
**Flk2 (total 16 sites in mouse and 34 sites in human)**		
6th site	32681 to 32690	conserved
**Cited2 (total 4 sites in mouse and 5 sites in human)**		
2nd site	−1236 to −1227	conserved
**Id1 (4 sites in mouse and 1 site in human)**		
4th site	5362 to 5371	conserved* Barham B et al., (2008) Nucleic Acids Research, 36:3802–3818
**Satb1 (total 31 sites in mouse and 47 sites in human)**		
1ST site	–967 to –958	conserved
3rd site	7188 to 7197	conserved
5th site	9399 to 9408	conserved
9th site	18291 to 18300	conserved
14th site	43276 to 43285	conserved
24th site	75292 to 75301	conserved
27th site	86572 to 86581	conserved
29th site	89174 to 89183	conserved

Notes: Gene sequence from each species was obtained from http://useeast.ensembl.org including 5000bp upstream and 5000bp downstream sequence. Stat5 binding sites (TTCnnnGAA) were found in the gene sequence using Clone Manager Suite software. Each gene sequence from mouse and human was aligned by global comparison with the Clone Manager Suite. The positions of conserved sites are listed in the table.

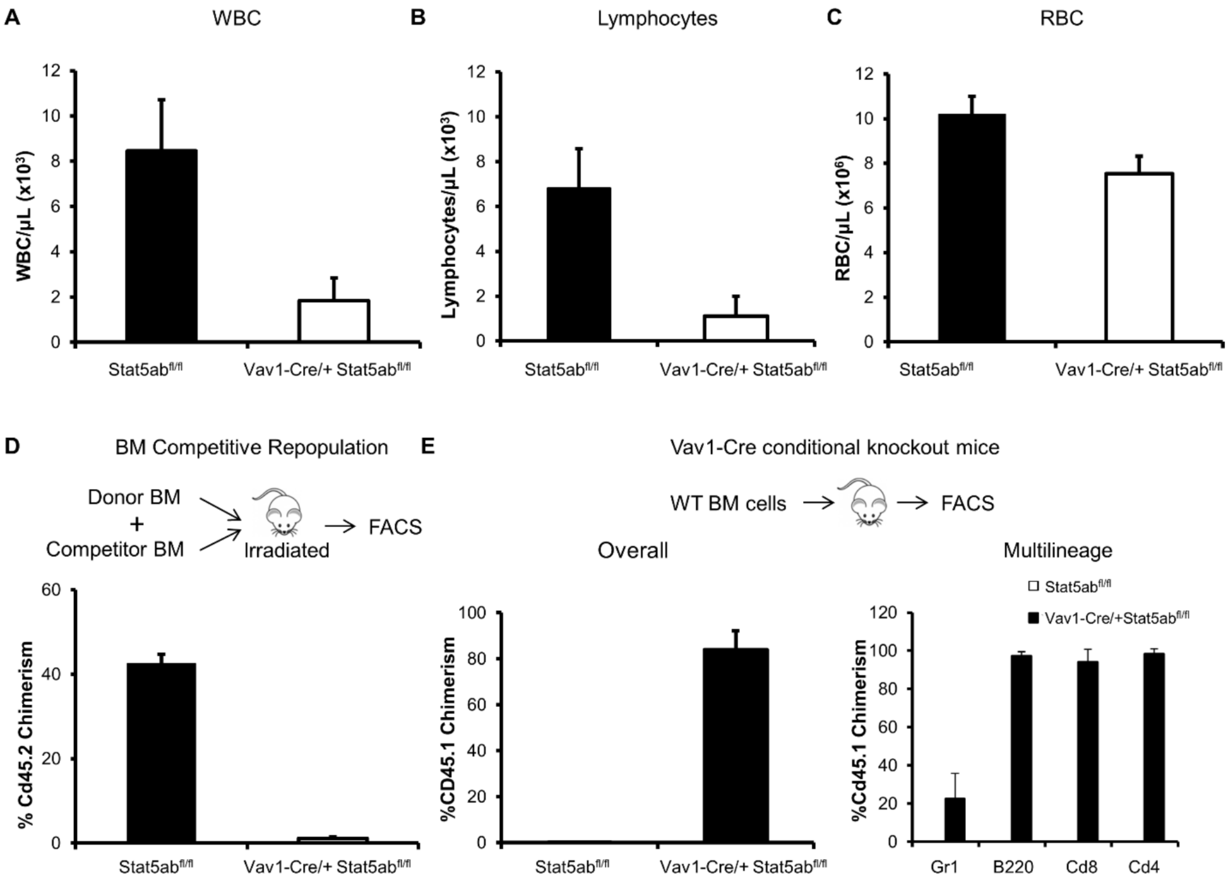

Supplementary Figure S1: Reduced hematology and competitive repopulation activity of viable adult Stat5 conditional knockout mice. A–C. Peripheral blood hematology of Stat5ab$^{fl/fl}$ and Vav1-Cre+/Stat5ab$^{fl/fl}$ mice at the age of 8–12 weeks old. Data presented are the average of Stat5ab$^{fl/fl}$ (n = 27) and Vav1-Cre+/Stat5ab$^{fl/fl}$ (n = 17) mice. **D.** Percentage of donor CD45.2 chimerism for competitive bone marrow transplantation 16 weeks after transplantation. Bone marrow cells from either Stat5ab$^{fl/fl}$ or Vav1-Cre+/Stat5ab$^{fl/fl}$ were mixed with BoyJ bone marrow cells at the ratio of 1:1 and transplanted into lethally irradiated C57BL/6 x BoyJ F1 recipients. Results are average of two independent transplantation experiments (total 10 recipients per group). **E.** Vav1-Cre/+Stat5ab$^{fl/fl}$ (n = 5) were injected with donor cells in the absence of irradiation. Recipient mice (CD45.2) were transplanted with 5 million CD45.1 positive bone marrow cells under non-ablative conditions as described(21). The percentage of donor chimerism on overall or each lineage was measured 16 weeks after the transplantation.

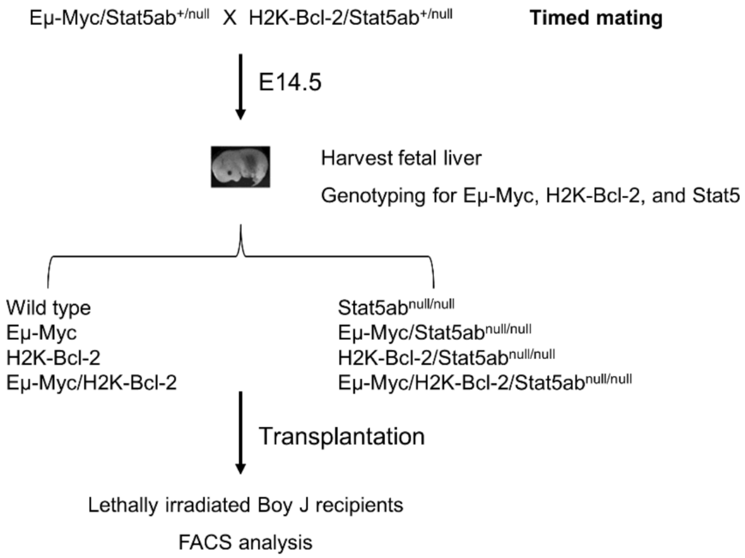

Supplementary Figure S2: Experimental design for fetal liver transplantation experiments to generate lymphoid malignancy. Both Eμ-Myc/Stat5ab$^{+/null}$ or H2K-Bcl-2/Stat5ab$^{+/null}$ were maintained as from two separate mouse breeding colonies with either transgene kept as a single allele. Since Eμ-Myc mice develop spontaneous lymphoma within 3–5 months, a relatively large mouse colony and extra care was needed to maintain this colony in order to obtain enough mice for setting up timed matings. Fetal liver harvest, genotyping, and transplantation were carried out on the same day.

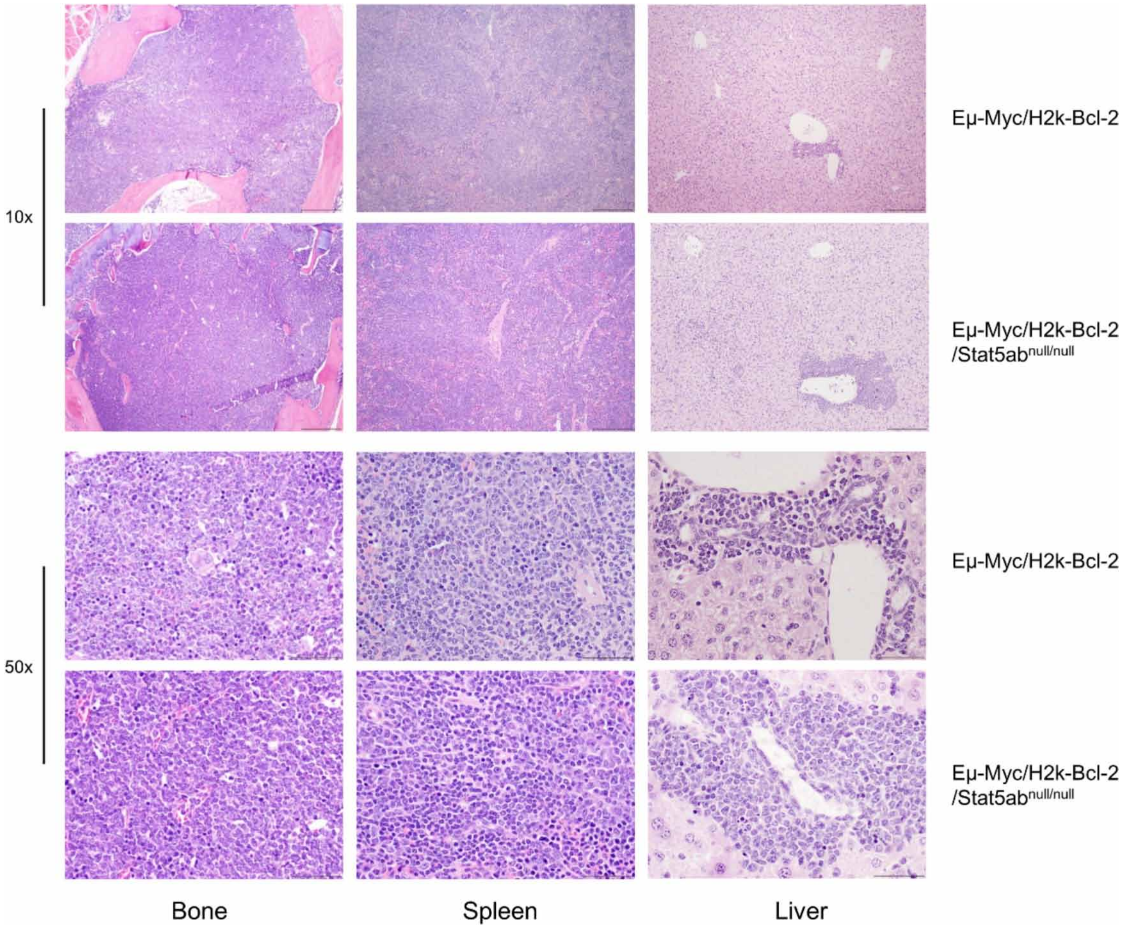

Supplementary Figure S3: Histopathology of B-ALL mouse model. E14.5 fetal liver cells (Eμ-Myc/H2K-Bcl-2, Eμ-Myc/H2K-Bcl-2/Stat5ab$^{null/null}$) were transplanted into lethally irradiated BoyJ recipient mice at the ratio of 1 donor fetal liver to 5 recipient mice. Tissues from mice that underwent transplantation were collected and formalin-fixed, and histologic sections were stained with hematoxylin and eosin. Representative sections of bone, spleen and liver are shown. The histology was performed at the end of the survival curve with mice clearly showing signs of sickness. Images were taken using an Olympus Model U-D03 microscope and a mounted Olympus DP21 digital camera. The software for image acquisition was Olympus CellSens Standard. Image processing was done in Adobe Photoshop. The size bar on 10x images is 200 μm and the size bar on 50x images is 50 μm. Both wild-type and knockout mice showed marrow replacement by a confluent sheet of immature mononuclear cells, consistent with leukemic blasts. Erythropoiesis and megakaryopoiesis was decreased. In the spleen there was an increased infiltrate by immature mononuclear cells in the white pulp and red pulp. Background extramedullary hematopoiesis was also present in the liver, where peri-portal and peri-venous infiltrates were observed compared to the wild-type. Hepatic parenchymal infiltrates were also present regardless of genotype. Both wild-type and knockout mice showed marrow replacement by a confluent sheet of immature mononuclear cells, consistent with leukemic blasts. Erythropoiesis and megakaryopoiesis was decreased. In the spleen there was an increased infiltrate by immature mononuclear cells in the white pulp and red pulp. Background extramedullary hematopoiesis was also present in the liver, where peri-portal and peri-venous infiltrates were observed compared to the wild-type. Hepatic parenchymal infiltrates were also present regardless of genotype.
